# Supplementary material for: Are carbon emissions trading and green financial instruments synergistic? -Comprehensive quantitative research based on content analysis
Source: PLoS One. 2024 Mar 7;19(3):e0298601. doi: 10.1371/journal.pone.0298601 (PMC10919601; doi:10.1371/journal.pone.0298601)
Supplement: S1 Data — (ZIP) [file pone.0298601.s001.zip › Data/地级市Z_F工作报告.zip!/┴╔─■/│»╤⌠╩╨/└┤╫╘╣1⁄2╓┌║┼í╛┬φ┐╦╩2╛▌═°í┐_2016.docx]

各位代表：

现在，我代表市人民政府向大会报告工作，请予审议，并请各位政协委员和其他列席人员提出意见。

一、“十二五”时期及2015年工作回顾

“十二五”时期，是朝阳发展极不平凡的五年。在省委、省政府和市委的正确领导下，我们认真落实党的十八大和十八届三中、四中、五中全会精神，抢抓新一轮东北振兴和突破辽西北等重大机遇，积极应对前所未有的困难和考验，开拓创新，真抓实干，全市改革开放和经济社会发展取得了新进展。

主动转变发展方式，积极探索经济转型之路。随着经济发展进入新常态，我市“一铁独大、低端主导”的经济结构和长期积累的体制机制弊端日益凸显。为实现转型发展，我们坚持问题导向，不断理清发展思路，坚定不移地实施“五大战略”，深入推进“五个一工程”，加快建设“五大基地”，积极培育工业“五大主导产业”，找到了符合朝阳实际的科学发展道路。

改革开放和体制机制创新不断深化。加快实施以简政放权为核心的行政审批制度改革，园（景）区、农村土地流转、商事制度、财税、金融、价格等领域改革扎实推进。坚持不懈地抓好招商引资和对外开放，出口创汇年均增长18.6%。

结构调整深入展开，发展短板逐渐弥补。现代农业迈出新步伐，设施农业扩量提质增效工程稳步推进，200万亩节水滴灌全面建成；工业结构调整不断深入，冶金业逐步调优，汽车及零部件、农产品及食品加工、能源及新能源、建材及设备制造等产业加速成长；服务业对经济增长的贡献率明显提高，占GDP比重达47.2%。地区生产总值年均增长6.7%。

生态环境和城乡面貌日益改善。凌河治理、城区集中供热、500万亩荒山绿化、草原沙化治理、人工影响天气等工程取得重大进展，我市成功晋升全国绿化模范城、省级环保模范城。新型城镇化步伐不断加快，常住人口城镇化率达45%。京沈客专朝阳段启动建设，朝阳至北京、上海、大连、天津航线陆续开通，外环路、燕白滨河路、文明路等建成投入使用。

民生保障持续推进，群众生活水平明显提高。累计投入社会民生资金614.5亿元。城镇和农村常住居民人均可支配收入分别增长64.2%和71.5%。社会保障体系逐步完善，社会事业全面进步，平安朝阳、法治朝阳建设深入开展，老旧小区改造、十分钟便民服务圈等重大民生工程扎实推进，人民群众有了更多的获得感。

各位代表，刚刚过去的2015年，受宏观环境和多重复杂矛盾交织等影响，朝阳经济持续下行，一系列新问题新挑战接踵而至，发展遇到前所未有的困难。面对困难和挑战，我们变压力为动力，迎难而上，奋力拼搏，取得了来之不易的工作成绩。与做实2014年基础数据相比，预计完成地区生产总值780亿元，下降1%；固定资产投资500亿元，增长4.9%；公共财政预算收入54.7亿元，下降25.5%；社会消费品零售总额420亿元，增长11%。城镇和农村常住居民人均可支配收入分别实现21283元和10535元，增长8.4%和8%。民生支出占财政支出比重达75%以上。

一年来，我们着力适应经济发展新常态，努力研究新情况、解决新问题，集中抓了5件大事。

第一，深入贯彻落实中央和省各项重大决策，把发展路子走对走好走实。将“四个着力”“四个驱动”和培育“六个新增长点”落实到发展行动上，制定实施意见，细化目标、责任和任务，形成了一系列实打实的工作方案，并以“钉钉子”精神认真抓好落实，使这些新理念、新举措成为推动朝阳振兴发展的行动纲领和重要抓手。

第二，科学编制“十三五”规划，谋划发展新蓝图。根据市委的建议，我们提早动手，深入调研，历时15个月完成了规划编制。市政府常务会议逐一审议了7个县（市）区发展规划和22个部门专项规划。在充分征求意见的基础上，形成了《朝阳市国民经济和社会发展第十三个五年规划纲要》，提请本次大会审议。

第三，全面推进“五个一工程”建设。按照新体制新机制搭建了发展平台，实行新的财政管理体制和行政审批制度，封闭管理运行。通过6次千人大会和系列现场会的强势推动，各园（景）区规划编制、组织机构搭建基本完成，基础设施建设和项目引进取得明显成效，加快发展的新动能迅速聚集。国电热电联产、东锆新材料开工建设，一汽凌源整车、荣富陶瓷等批量生产，航天凌河重卡、中信化石产业园、绿探新能源等重点项目实现落地，与中国供销集团、中粮集团、北京新发地等合作项目加快推进。双塔万达广场从洽谈到落地仅用4个月时间，创造了万达广场全国项目的最快速度。5个县级工业开发区全部纳入省级开发区进行预管理；朝阳县、建平、喀左服务业集聚区进入省重点扶持行列，7个县（市）区实现省级服务业集聚区全覆盖；北票五间房等5个新市镇均被列为全省宜居乡村试点镇；龙城、喀左农产品加工园区被列为省政府重点支持园区。

第四，强力启动矿山矿业综合整治。针对矿业经济下滑的实际，我们以“壮士断腕”的决心，全面启动了矿业整顿、采石场整治、打击矿产资源违法犯罪三大专项行动，依法关闭矿山和采石场128处，侦办涉矿犯罪37起，依法收缴一大批涉案物资，矿业整顿首战告捷。

第五，打响脱贫攻坚战。对农村贫困户进行精准识别，实行动态调整，做到有进有出。同时为每个贫困户建档立卡，逐一分析贫困程度和致贫原因，对症下药，研究制定具体脱贫办法，做到因户因人施策、精准扶贫、精准脱贫。

一年来，我们还统筹稳增长、促改革、调结构、惠民生、防风险，重点做了以下工作。

一是加快推进改革开放。积极争创全国简政放权试点地区、全省开发区审批制度改革试点市，全面公布部门权责清单和收费清单，审批前置要件精简率达67%，基本建设项目审批时限压缩到25个工作日以内。“三证合一”“一照一码”登记制度改革全面完成。国资国企改革取得新进展，市属经营性国有资产集中移交基本完成。富有成效地组织了15次京津冀等地大型招商活动；积极扩大面向俄罗斯等“一带一路”沿线国家的开放合作，实现外贸出口8.13亿美元，增长7.6%，增幅列全省第一位。

二是推动经济稳定增长。大力开展项目建设年活动，制定出台了“稳增长20条”和一系列房地产促销政策，对县域经济和园（景）区进行多轮次现场办公，加大停产半停产企业帮扶力度。启动实施中国制造2025朝阳行动纲要，推进工业“五大主导产业”建设。与中科院金属研究所签订了全面科技合作协议，凌钢金属防腐涂层项目正式签约；朝柴在凌钢托管后逐渐焕发生机；浪马轮胎在市场下滑的形势下产销量突破200万套；新能源汽车产业取得新进展，江苏天洋新能源专用车项目正式签约，佳翔、皖宁低速电动车开始生产。充分利用棚改政策化解房地产库存，完成棚户区改造1.5万户，消化存量房1.2万套。积极培育养老、健康、体育等新消费热点，扩大消费需求。加大金融支持力度，新增本外币贷款87.8亿元，增长10.6%，帮助企业实现直接融资56亿元。凌钢成功实现股票定向增发20亿元，全国最大的民营担保机构——瀚华金控落户朝阳。朝阳银行存贷款业务强劲增长，经营效益大幅提高，锦州分行获省银监局批复筹建。

三是大力发展现代农业。克服连续严重旱灾影响，新增节水滴灌面积71.4万亩，粮食产量实现39.3亿斤。扎实推进设施农业扩量提质增效工程，设施农业实现总产量547.6万吨、产值159亿元。农产品加工园区建设取得较大进展，新增农业产业化重点龙头企业25家。农产品现代流通体系建设扎实推进，市供销集团成功组建。畜牧业产值首次突破200亿元。

四是推进服务业加快发展。“3+7”景区发展格局全面拉开，凤凰山基础设施完善工程、鸟化石景区提升工程、牛河梁生态农庄等扎实推进，龙凤山、龙源景区晋升国家4A级景区，喀左紫砂壶获全国旅游特色产品银奖。市电子商务产业园被评为全省电子商务集聚示范区，凌源被评为全国农村电子商务示范县，北方矿产品和朝阳农品等电子商务平台投入运营。城乡商品市场和现代物流体系建设加快推进，我市被确定为全省肉菜流通追溯体系试点市。

五是开展大众创业、万众创新活动。在全省率先启动“双创”工作，出台36条扶持政策，建设一批高标准创新创业孵化基地，掀起了“大众创业、草根创业”浪潮，带动一批新产业、新业态发展。喀左孵化基地被评为省级示范基地，科技大厦创新工坊投入运营，“天佑紫砂”、“城市蜗牛”等朝阳籍双创品牌不断涌现，新增创业企业2400多家、个体工商户9000多户，带动就业1.9万人。

六是加快推进城乡建设。燕凌大桥竣工通车，文明路公铁立交桥、朝巴线改移启动建设，京沈客专朝阳段、辽西北供水及配套工程进展顺利。城市东部排污主干网竣工运行，滨河路、文明路绿化全面完成。市容环境集中整治深入开展，城市管理水平不断提升。美丽乡村建设、农村危房改造扎实推进。

七是努力改善生态环境。城区环城绿化带、凤凰山迎城面采石场植被恢复工程加快实施，完成荒山造林98.1万亩，水土流失综合治理76.8万亩，草原沙化治理52万亩。凌河流域水质稳定达标，沿河城乡防洪及生态景观建设取得新进展。通过环境综合整治“百日行动”等系列综合措施，雾霾治理取得扎实成效，全年空气质量综合指数在全省排名第一。

八是化解债务风险和历史遗留问题。积极采取政府债券发行、置换债券、建立偿债准备金等措施，加强政府债务管理，确保到期债务按期偿还。努力化解各种沉积问题，一批企业无房产证、征迁居民回迁难、建筑工地手续不全等问题得到及时解决。

九是着力保障和改善民生。十大重点民生工程、35件民生实事全部落实。其中，就业保持平稳，城镇登记失业率为3.48%。各类社会保险覆盖面不断扩大，基本医疗保险实现市级统筹。完成老旧小区改造120万平方米，8.7万农村人口饮水安全问题得到解决。十分钟便民服务圈建设扎实推进，社区老年人日间照料室达到131个，城区65%以上老旧弃管小区有了物业管理，“智慧社区”建设等工作取得明显进展。

十是积极发展社会事业，维护社会和谐稳定。新建改扩建15所标准化幼儿园，完成120所义务教育学校标准化建设，普通高中优质特色建设取得新成果，现代职业教育加快发展。9家县级公立医院全面取消药品加成，90%以上乡镇实现了政府办卫生院目标。世界地质公园申报已通过专家委员会评审，积极推进博物馆城建设，成功举办“书香朝阳”全民阅读、凌河沿岸群众文化活动带暨群众文化节、非物质文化遗产展示展演、全民健身节等活动。加强闲置土地处置，国土资源管理水平和保障能力不断提高。社会治安防控体系建设得到加强，维稳、安保、道路交通秩序整治、火灾隐患排查取得明显成效，人民调解、社区矫正、法律援助取得新进展。成功侦破公安部督办的2015年1号专案，追缴文物2063件（套），其中一级文物248件（套）。信访改革成效显现，信访总量大幅下降。持续开展隐患排查和整改，安全生产形势总体平稳。民族团结进步事业不断发展，宗教事务管理水平逐步提升。双拥创建扎实推进，军地融合发展呈现新局面。国防动员、人防和民兵预备役建设进一步加强，食药监、住房公积金、邮政、档案、应急管理、国家安全、援疆援青、外事侨务以及老年人、妇女儿童、残疾人权益保障等工作也取得了新成绩。

过去一年，我们自觉接受人大及其常委会的法律监督和工作监督，主动接受政协的民主监督，共办理人大代表议案2件、建议71件和政协提案173件，满意率均达到100%。扎实开展“三严三实”专题教育，健全完善政府绩效管理、法律顾问制度，规范涉企执法行为，加大行政监察和审计监督力度，严肃查处了一批违纪违法案件。

这些成绩的取得来之不易，是省委、省政府和市委坚强领导、正确决策的结果，是市人大、市政协以及社会各界大力支持、鼎力相助的结果，是全市人民团结一心、共同努力的结果。在此，我代表市政府，向各个岗位上辛勤付出的全市各族人民，向给予我们监督支持的人大代表、政协委员、各民主党派、工商联、无党派及社会各界人士，向参与朝阳发展建设的驻朝中省直单位、解放军指战员和武警官兵，表示诚挚的感谢！

各位代表，当前我市发展也面临着新的困难和问题。一是工业下行压力持续增大。去年规模以上工业增加值下降17.5%，工业品价格和企业利润持续下滑，工业生产者出厂价格指数（PPI）连续50个月负增长，铁选冶金企业出现大面积亏损和停产，仅资源税减收就达11.8亿元。二是财政收支矛盾突出。财政收入一直负增长，而刚性支出不减反增，财政收支平衡面临巨大压力。三是新增长点不足。支柱产业急剧下滑，新落地项目大多处于建设期，尚未形成贡献。四是潜在风险增多。政府债务进入偿还高峰期，企业效益下滑，养老、工伤保险等社保基金征缴难度越来越大。随着经济下行，就业压力也逐渐显现。另外，一些干部没有摆脱“速度情结”和“换挡焦虑”的思维定势，对新常态认识不足、被动适应，能力还存在欠缺。少数部门和公职人员不担当、不作为、不勤不廉现象仍然存在，一些腐败案件损害了政府形象。

对这些困难和问题，我们将高度重视，采取有力措施加以解决。

二、“十三五”时期主要目标和任务

各位代表，“十三五”时期是全面建成小康社会的决胜期。当前，经济发展进入新常态，速度在换挡，发展方式在转变，增长动力机制在发生重大转换，朝阳振兴发展到了滚石上山、爬坡过坎的关键阶段。我们要把握好这一阶段性特征，进一步增强适应能力，坚定发展信心。

我们要看到，我国宏观经济仍处于“四个没有变”的战略机遇期，朝阳经济总体向好的基本面没有变，结构优化调整的前进态势没有变，这将坚定我们发展的信念和底气。我们要看到，当前我市面临新一轮东北振兴和突破辽西北等重大政策机遇，改革开放的红利正在释放，区位条件和发展基础正在改善，后发优势更为明显。我们要看到，在经历了调整和阵痛之后，朝阳发展已形成了明晰思路，特别是“五个一工程”实施以来，一批稳增长、调结构项目正加快孕育并开始破茧而出，一些新的经济增长点和发展动力正加快形成。我们要看到，朝阳政治生态在好转，干部的精气神在提振，干事创业的动能在集聚，这些都将成为加快发展的积极因素。只要我们坚定信心，苦干实干，就一定能战胜前进道路上的艰难险阻，夺取全面建成小康社会的最后胜利！

“十三五”时期发展的主要目标是：地区生产总值年均增长7%，城乡居民人均可支配收入与经济增长同步，确保到2020年，地区生产总值和城乡居民人均可支配收入按可比口径比2010年翻一番。公共财政预算收入年均增长6%，固定资产投资年均增长7.5%，社会消费品零售总额年均增长8%，外贸出口年均增长8%。5个贫困县和413个贫困村全部摘帽，17.16万贫困人口全部脱贫。

今后五年，我们要按照“四个全面”战略布局，把“五大发展理念”落实到工作实践中，把“四个着力”贯穿到发展行动上，坚持“四个驱动”共同发力，培育和壮大“六个新增长点”，积极推进供给侧结构性改革，努力为朝阳新一轮振兴发展注入新动力、拓展新空间。

——形成更具活力的体制机制。进一步完善“五个一工程”体制机制，大力实施以简政放权、审批制度改革为重点的行政体制改革，努力把朝阳建成全省乃至全国行政审批效率最高的地区之一。坚持市场化改革方向，深化国资国企、农村、开放、财税、投资、价格等领域改革。

——持之以恒推进产业结构调整。保持战略定力，增强战略耐心，加快“五大基地”“五个一工程”建设，培育工业“五大主导产业”，推进制造业提档升级，大力发展现代农业、现代服务业和文化旅游产业，实现战略新兴产业和传统制造业并驾齐驱、现代服务业和传统服务业相互促进、信息化和工业化深度融合。

——培育带有朝阳特色的创业创新。发挥“双创”的拉动乘数效应，推动新技术、新产业、新业态蓬勃发展，努力在新能源汽车、新材料、新能源电器、环保产业等方面实现突破。深入开展大众创业、万众创新活动，激发全社会的创业热情和活力。

——建设惠及340万人民的全面小康。以更精准的举措、更超常规的力度，打赢脱贫攻坚战，让所有贫困村、贫困户真正拔掉“穷根”，决不落下一村一人。全力解决好人民群众的就业、社保、教育、医疗、环境等问题，补齐全面小康社会在民生领域的短板，使民生改善和经济发展有效对接、相得益彰。

——打造生态文明更加彰显的美丽朝阳。大力实施生态立市战略，把绿色发展理念融入经济社会发展中，继续抓好青山、碧水、蓝天、沃土四大生态工程，推进矿山矿业综合整顿，加快生态环境修复，让朝阳蓝天常驻、空气常清、绿水长流、青山常翠。

三、2016年工作安排

各位代表，今年是“十三五”规划开局之年。当前，宏观经济下滑尚未触底，我市铁选冶金业、建筑业和房地产业经受的冲击还在延续，一些长期积累的深层次矛盾在经济减速下更加水落石出。这些问题不是短期形成的，实现新旧动能转换将是一个充满阵痛、十分艰难的过程。我们必须卧薪尝胆，勇于接受改革和结构调整带来的阵痛，积极把握好稳增长和调结构的平衡，以知难而进的勇气和战胜困难的决心，全力抓好经济社会发展各项工作。

推进供给侧结构性改革是今年经济工作的一条主线，是解决经济发展问题的根本之道。其目的是实现经济供需两侧在新常态下的调整和平衡，核心是优化存量、做大增量、主动减量。我们一定要瞄准靶心，对准病灶，精准发力，集中抓好去产能、去库存、去杠杆、降成本、补短板“五大任务”，努力提高经济发展的质量和效益。

今年全市经济社会发展主要预期目标是：地区生产总值增长5%左右；公共财政预算收入增长2%左右；固定资产投资增长6%左右；社会消费品零售总额增长8%左右；城镇登记失业率控制在4.5%以内；城乡居民可支配收入增长与经济发展同步。

（一）努力保持经济稳定增长

稳住经济运行仍是今年经济工作的重中之重。我们要拿出更有效的措施，深挖各种增长潜力，提高稳增长的质量和效益。

开展项目建设年活动。把“五个一工程”建设重心向引项目、落项目、建项目聚焦，争取一批重点项目尽快签约落地、开工建设、投产达效。提高投资的有效性和精准性，保证项目建设质量和效益，实施亿元以上重点项目100个，完成投资400亿元以上，每个园（景）区都要争取有一个10亿元以上核心项目开工建设。在交通、水利、能源、生态、城市管网、扶贫开发等领域，推广政府与社会资本合作机制。通过整合专项资金、实施“拨改投”等方式，建立产业发展引导基金，破解工业发展和项目建设的资金瓶颈问题。

抓好企业运行。坚持问题导向，深入开展走进企业走进项目帮扶活动，保企业、保开工、保正常运转。积极推进资源型企业优胜劣汰，整合升级一批深加工企业，清理一批僵尸企业，将有效资源向重点企业倾斜。实施助力企业降本增效行动，打好降低交易、人工、财务、物流成本和税费、社会保险费、电费负担等“组合拳”。规模以上工业增加值增长3%左右。

多措并举扩大消费。顺势发展促进消费的新业态，推进现代流通体系建设。支持发展旅游休闲、教育文体、养老健康、家政服务等新的消费热点，不断释放新消费、创造新供给、形成新动力。

进一步抓好城镇棚户区、农村危房改造及配套基础设施建设，通过棚改政策消化、户籍制度改革、鼓励开发企业调整营销策略等措施，化解房地产库存。

（二）大力推进产业结构调整升级

坚持增量调结构、“加减乘除”一起做，加快建设“五大基地”、打造工业“五大主导产业”。

抓好传统产业转型升级。支持凌钢实施“普转特”，推进金属防腐涂层、天翼国基高纯合金等项目；支持鞍钢朝阳公司实施产品结构调整和升级；支持金麟高纯铁粉、新都黄金搬迁改造、北方兄弟资源不锈钢、庆昇粉末冶金、金锐钒钛等高品质冶金项目建设，加快调优冶金业。抓好一汽凌源新厂区、航天凌河重卡、浪马轮胎扩能、朝柴国五发动机等项目，做大汽车及零部件产业；依托航天科技、天洋集团及立塬公司打造新能源汽车产业园区，确保取得实质性突破。积极推进国电热电联产、立塬新能源、百纳超级电容器、航天长峰电源等项目，大力发展风电和光伏发电，不断壮大能源产业。抓好朝重、重机、黑猫炭黑搬迁改造以及华潍膨润土、煜梽新型建材、中智力拓镀膜玻璃深加工、赫源陶瓷等项目，做强建材及设备制造业。继续实施矿山矿业综合整顿，年底前矿山整合到100处以内，采石场减少到40处以内。

加快培育战略性新兴产业和高新技术产业。全面实施中国制造2025朝阳行动纲要，开展智能制造和智能服务试点示范，积极推进佛瑞达包装新材料智能生产线、赛克瑞斯机器人等项目，加快制造强市建设。协调推进新能源、新材料、节能环保、电子信息、精细化工产业发展。

大力发展现代农业。抓好农产品加工园区和重点产业化项目建设，深入实施设施农业扩量提质增效工程，加强农产品区域公用品牌建设，继续扩大节水滴灌面积，使朝阳农产品品种、质量更加契合市场需求。

加快发展现代服务业。大力实施文化旅游牵动战略，抓好重点景区基础设施完善、战略投资者引进、营销体系建设、景区资源联动等工作，打造留得住游客的休闲度假区、旅游产业集聚区。加快服务业集聚区建设，积极推进万达广场、天马时代广场、河东凤凰商城、居然之家等项目建设。促进传统服务业提质增效升级，实施“互联网+”行动计划，大力发展电子商务、现代物流等新业态。推进农产品现代物流体系建设，积极谋划建设蔬菜、杂粮大型专业市场，做好肉菜流通追溯体系试点工作。抓好产业金融服务体系建设，利用多层次资本市场进行融资。

（三）深入推进改革

抓住经济发展的结构性矛盾和深层次问题，大力推进结构性改革，不断释放改革红利、激发动力活力。

深化“简政放权、放管结合、优化服务”改革，进一步增强放权的协同性，提高监管的有效性，强化服务的便利性。做好全国简政放权试点、全省开发区审批制度改革试点争取工作，并准备全面铺开推进，通过市县联动，大幅度减少行政审批事项，大幅度减少审批前置要件，大幅度减少涉企收费，使中介机构与政府部门真正脱钩。发挥政府部门权责清单约束作用，实行随机抽查制度。抓好投资领域改革，加强公共行政服务中心和网上审批系统建设，优化项目审批流程。推动财税体制改革，促进依法理财。深化商事制度改革，进一步简化注册资本登记。

把“五个一工程”作为深化改革的重要平台，健全完善管理体制机制，搞好选人用人、分配和考评机制创新，进一步激发活力。继续取消下放一批行政审批事项，促进各县（市）区和园（景）区做好承接工作。

实施市属经营性国有资产分类改革，整合优良资产，组建一批具有活力和市场竞争力的企业集团，盘活存量，放大国有资本功能。

推进共营制新型农业经营体系建设试点，引导土地承包经营权有序流转，促进土地规模化经营、专业化生产、社会化服务和产业化发展。进一步扩大农村土地承包经营权确权颁证范围。加快推进供销社综合改革和农村信用社改革。

稳妥推进事业单位分类改革。做好公务用车制度改革。

（四）加快推动创新发展

坚持引进消化吸收再创新，加强与省内外科研院所、大专院校战略合作，进一步完善产学研协同创新机制，实施科技成果转化项目50个以上。支持企业引进新技术、开发新产品，加快推进光达化工耐低温电解液、九通环保摩擦材料等项目，促进传统产业转型升级。高新技术企业营业总收入增长10%。

积极推动大众创业、万众创新。抓好创新创业大学、众创空间和创业工坊、企业孵化器建设，加速形成“三位一体”的创新创业模式。加大新兴产业、小微企业支持力度，形成多点开花的民营经济发展局面。

以“五个一工程”建设需求为依托，制定相关政策，培育创新人才队伍，拓宽人才引进渠道。调整职业教育结构，提高专业与产业吻合度，抓好创新型人才培养。

（五）统筹城乡协调发展

坚持产城融合，加快新型城镇化进程。积极稳妥推进各新城新区基础设施、产业支撑和配套功能完善，提升承载能力。抓好新市镇试点，巩固发展“产业园区、中心街区、美丽乡村”三位一体建设，每个县（市）区启动建设2—3个重点镇和特色镇。新实施棚户区改造7494户，完成城中村改造100万平方米、小城镇建设100万平方米，进一步提高户籍人口城镇化率。

改善城区环境和功能，全面启动环城生态廊道建设工程，积极打造“海绵城市”“智慧城市”。完成老旧小区改造60万平方米、弃管小区物业扩面400万平方米。

加快宜居乡村建设，继续开展一事一议村内道路和美丽乡村示范村建设，逐步实现村路硬化、村庄亮化、村容美化、村屯绿化。

推进京沈客专至赤峰、盘锦两条连接线，以及锦承线朝阳段、叶赤线扩能改造工程征地拆迁工作，规划建设市综合客运枢纽工程，实施朝巴线改移、新市镇与主干道相连道路建设项目，启动全国绿色交通试点市建设。推进农网改造升级工程。配合省里完成辽西北供水二期工程相关工作，推进市净水厂及配套管网建设。

（六）加强生态保护和环境治理

实施青山工程。抓好闭坑矿山生态治理，推进环城绿化带建设，完成凤凰山迎城面采石场覆绿工程。实现造林绿化60万亩、水土保持生态治理70万亩。

实施碧水工程。加强凌河水源保护，以“三带”规划为核心，筑牢“水质安全、行洪安全、生态安全”底线，提高依法治河和精细化管理水平。确保污水处理厂稳定达标运行，抓好集中式饮用水源地保护、中水回用、水质达标工作。

实施蓝天工程。出重拳防治大气污染，大力清理整顿环保违规建设项目，拆除城市建成区内10吨以下燃煤锅炉，加快中心城区北部及各县城集中供热改造，做好淘汰黄标车、供应国五标准车用汽柴油、扬尘整治、机动车尾气污染防治等工作，确保空气质量综合指数保持在全省前列。

实施沃土工程。积极推广保护性耕作、土地深松和化肥减施。做好土壤环境监测工作，抓好面源污染治理。

（七）进一步提升对外开放水平

积极融入京津冀协同发展和“一带一路”战略，主动参与中蒙俄经济走廊建设，开拓发展新空间。

强力推进面向京津冀的“五个一工程”重大项目和主导产业招商、面向上海等长三角地区的金融和科技招商。坚持与产业结构调整和转型升级相结合，深化与央企和行业500强企业合作，进一步提升项目质量。

发挥资源优势，加强出口基地建设，扶持出口企业发展，形成以大企业为牵动、中小企业共同抓出口的局面。每个县（市）区至少培育一家外贸综合服务企业。出口创汇增长5%以上。

积极推进钢材、汽车零部件、农产品、矿山设备等优势资源和富余产能走出去，扩大对“一带一路”沿线国家的开放合作。支持企业开展境外工程承包，做好境外科技型企业并购工作。

（八）全力保障和改善民生

实施脱贫攻坚工程，精准分析致贫原因，集中整合各类资金和项目，采取产业扶持一批、培训就业一批、医疗救助一批、教育资助一批、移民搬迁一批、低保兜底一批的办法，完成5.25万贫困人口脱贫任务。

实施更加积极的就业政策，促进大学生、返乡农民工及就业困难人员创业就业，落实好援企稳岗等政策，实现城镇新就业2.6万人。

推行“五险合一”参保模式，加大基金征缴力度，确保各项社会保险待遇按时足额发放。推进机关事业单位养老保险制度改革。完善社会救助体系，提高城乡低保、大病救助、社会福利和救灾救济水平。深入推进十分钟便民服务圈建设，不断完善城市功能。

均衡发展社会事业。新建和改扩建农村标准化幼儿园10所。启动全国义务教育发展基本均衡县创建工作，推动普通高中教育优质特色发展，加快朝阳师专办学转型升级。积极发展民族教育、特殊教育和民办教育。深入推进医药卫生体制改革，深化县级公立医院综合配套改革，推动“三医联动”。继续抓好市妇儿医院和公共卫生服务综合楼项目建设。落实全面放开二孩政策，不断提高优生优育水平。繁荣精品艺术创作，建设“书香朝阳”，完成送戏下乡400场。抓好世界地质公园申报和牛河梁遗址“申遗”工作。加快市博物馆、规划馆建设，确保年内投入使用。推进群众体育和竞技体育协调发展。

积极创新社会治理。加强应急管理，强化安全生产、食品药品安全监管，严防发生重特大事故。推进消防应急救援队伍和基础设施建设，提高防灾救灾能力。启动“七五普法”，依法综合治理各类信访问题，从源头上化解社会矛盾。创新完善立体化社会治安防控体系，增强群众安全感。支持民族地区加快发展。大力开展“双拥”创建活动，抓好国防动员、国防教育，推进军民融合向深度拓展。支持工会、共青团、妇联等人民团体发挥桥梁纽带作用，保障妇幼权益，关心老龄事业，做好气象、防震、外事侨务、档案、人防、社科、残疾人等各项工作。

各位代表，推进法治、创新、高效和廉洁政府建设，是完成今年各项任务的重要保证。一是自觉尊法学法守法用法，建立健全决策合法性审查机制和政府法律顾问制度，把各项工作纳入法治轨道。主动接受人大及其常委会的法律监督、工作监督和政协的民主监督，认真执行人大及其常委会决议决定，积极落实“三个纳入”等加强人民政协协商民主新要求，做好意见、建议和政协提案办理工作。广泛听取民主党派、工商联、无党派人士和人民团体的意见建议，不断提高政府工作法治化、规范化水平。二是坚持法定职责必须为、法无授权不可为，讲诚信、懂规矩、守纪律，勇于负责，敢于担当，狠抓落实，增强政府的公信力和执行力。三是严格遵守中央八项规定精神，严格落实党风廉政建设“一岗双责”，严格执行《准则》和《条例》，努力做到忠诚、干净、担当。加强行政监察、审计监督，健全政府系统廉政风险防控体系，全面推进政务公开，强化对权力运行的监督和制约。四是加强目标管理，层层传导压力。健全完善工作实绩考核办法，对实绩突出的大力褒奖，对工作不力的约谈诫勉，对懒政怠政的追究责任，激发调动各方面的积极性，形成加快发展的强大合力。

各位代表，告别“十二五”，踏上新征程。让我们在市委的坚强领导下，凝聚全市各族人民的智慧和力量，抢抓机遇，开拓创新，扎实工作，努力实现“十三五”的精彩开局，在建设富裕文明和谐美丽新朝阳的道路上，谱写更加辉煌的篇章！
